# Cell-Free Seminal mRNA and MicroRNA Exist in Different Forms

**DOI:** 10.1371/journal.pone.0034566

**Published:** 2012-04-10

**Authors:** Honggang Li, Shiyun Huang, Cuicui Guo, Huangtao Guan, Chengliang Xiong

**Affiliations:** 1 Family Planning Research Institute/Center of Reproductive Medicine, Tongji Medical College, Huazhong University of Science and Technology, Wuhan, China; 2 Wuhan Tongji Reproductive Medicine Hospital, Wuhan, China; 3 Family Planning Department, Beijing Obstetrics and Gynecology Hospital, the Capital University of Medical Science, Beijing, China; University of Muenster, Germany

## Abstract

**Background:**

The great interest in cell-free mRNA, microRNA (miRNA) as molecular biomarkers for clinical applications, and as ‘signaling’ molecules for intercellular communication highlights the need to reveal their physical nature. Here this issue was explored in human cell-free seminal mRNA (cfs-mRNA) and miRNA (cfs-miRNA).

**Methodology/Principal Findings:**

Selected male reproductive organ-specific mRNAs, miRNAs, and piRNAs were quantified by quantitative real-time PCR in all experiments. While the stability of cfs-miRNA assessed by time-course analysis (up to 24 h at room temperature) was similar with cfs-mRNA, the reductive changes between cfs-miRNA and cfs-mRNA after filtration and Triton X-100 treatment on seminal plasma were very different, implying their different physical nature. Seminal microvesicles (SMVs) were then recovered and proportions of cfs-mRNA and cfs-miRNA within SMVs were quantified. The amounts of SMVs- sequestered cfs-mRNAs almost were the same as total cfs-mRNA, and were highly variable depending on the different sizes of SMVs. But most of cfs-miRNA was independent of SMVs and existed in the supernatant. The possible form of cfs-miRNA in the supernatant was further explored by filtration and protease K digestion. It passed through the 0.10-µm pore, but was degraded dramatically after intense protease K digestion.

**Conclusions/Significance:**

The predominant cfs-mRNA is contained in SMVs, while most cfs-miRNA is bound with protein complexes. Our data explained the stability of extracellular RNAs in human semen, and shed light on their origins and potential functions in male reproduction, and strategy of developing them as biomarkers of male reproductive system.

## Introduction

Cell-free RNAs, including mRNAs and miRNAs, are extracellular RNAs existing outside cells. In recent years, much interest has been focused on the potential diagnostic application and function of cell-free RNAs in human body fluids. Cell-free RNAs have been detected in many kinds of human body fluids and reported as promising biomarkers for various pathologic conditions, including cancer detection and prognosis [1], [2], prenatal diagnosis [3], [4], pregnancy monitoring [5], [6], tissue injury [7]–[9], cardiovascular diseases [10], metabolic disorders [11], and forensic identification [12], [13]. On the other hand, some recent reports have demonstrated the possible cell-cell communication function of extracellular mRNA and miRNA [14]–[18]. Extracellular miRNA may act like hormones [2].

Although the characterization, potential function and clinical application of cell-free RNAs in human body fluids have been demonstrated, relatively little is known about forms of their existence, which should be essential for the understanding their stability, origin, and mechanism for intercellular communication. The diagnostic and prognostic potential of cell-free RNAs as biomarkers relies mainly on their high stability, but RNA species are inherently labile and easily degraded in extracellular environments. To further spread the research and clinical applications of extracellular RNAs, it is important to understand the their very existence that could bring cellular RNAs into body fluids and protect them from degradation. On the other hand, the exploration of the physical nature of cell-free RNAs, which should be related with mechanisms of their secretion from, and entrance into cells, will shed light on their possible biological roles and mechanisms.

On the basis of the characteristics of cell-free seminal mRNA (cfs-mRNA), miRNA (cfs-miRNA) reported from our group [19] and others [20], the cfs-mRNA and cfs-miRNA appear to be potential noninvasive biomarkers for diseases of male reproductive organs and forensic identification [12], [13]. First, the concentration (average 1.75 mg/L [19] or higher [20]) of cell-free RNA in human seminal plasma is much higher than in other body fluids, also with high stability. Second, human seminal plasma is a mixture of fluids from the male reproductive organs, so it contains many tissue specific mRNAs and miRNAs of these organs. Moreover, because the cfs-mRNA and cfs-miRNA contain the RNA information of whole or bilateral male reproductive organs, it should be more representative than tissue biopsy, which is not easily applied and accepted for male reproductive organs.

As for the possible function of cfs-mRNA and cfs-miRNA, it is tempting to think that before ejaculation, extracellular RNAs stored in luminal fluids of male reproductive organs could commute between cells (maybe including sperm) and attribute to the gene expression, given that temporal and spatial regulation of gene expression in testis and epididymis is pivotal for normal spermatogenesis and sperm maturation. In sperm, RNA can be translated into proteins [21]–[23] and be delivered into oocyte [24], [25].

Some characteristics of human seminal plasma and male reproductive organs may attribute to the large amounts of RNAs in human seminal plasma, and to the forms of their existence. Human seminal plasma contains significant amounts of seminal microvesicles (SMVs) and proteins. SMVs are secreted from each male reproductive organ [26]–[28]. The epididymis and prostate release amounts of microvesicles into the glandular lumen via apocrine secretion, named epididymosome and prostasome [26], [28]; In the process of sperm maturation, sperm residual cytoplasm and cytoplasmic droplet are discarded from the nascent spermatozoa, partially via microvesicle budding. Moreover, during normal spermatogenesis, prominently germ cells undergo apoptosis, which may be important sources for both SMVs and cytoplasmic complexes releasing into luminal tubules [29]. Therefore, we hypothesize male reproductive organ-derived cell-free RNAs would be primarily sequestered within a variety of SMVs or cytoplasmic complexes in order to escape from degradation by extracellular RNases and be liberal present.

Although the presence of cfs-mRNA [19] and cfs-miRNA [20] was reported, and their potential applications and functions were proposed [12], [13], [19], [20], their physical nature remained unclear. In our previous study of the presence, integrity and stability of cfs-mRNA [19], we observed significant reduction of cfs-mRNA amounts after passing seminal plasma through filters (≤0.45 µm) or adding Triton X-100 into seminal plasma, whereas for cfs-miRNA no reduction or slight reduction was observed in our initial experiment (unpublished data) for the present study, implying different physical nature of cfs-mRNA and cfs-miRNA. Here we revealed the stability of cfs-miRNA, and explored the physical nature of cfs-mRNA and cfs-miRNA, by quantifying male reproductive organ-specific mRNAs, miRNAs, and piRNAs. To our knowledge, this represents the first report of the existing forms of cell-free RNAs in human seminal plasma. Very different forms of the existence of cfs-mRNA and cfs-miRNA were found, which should be responsible for their stability and help to spread their clinical applications, and shed light on their origins and possible roles in male reproduction.

## Materials and Methods

### Participant Recruitment and Seminal Plasma Isolation

The study was approved by the institutional review board at our facility: Reproductive Medicine Review Board of Tongji Medical College, and written informed consent was obtained from all volunteers included in this study. Clinical investigation was conducted according to the principles expressed in the Declaration of Helsinki.

The healthy participants (normozoospermic individuals) were selected from infertile couples who underwent assisted reproduction technology treatment for diagnosed female factor infertility, as described previously [19]. Vasectomized individuals were recruited from Qichun County (Hubei province, China) by local Family Planning Service Center. The mean age of the healthy participants and vasectomized individuals was 32 years (range, 26–42 years). The WHO's standard criteria [30] for normozoospermia was applied. All individuals had no family history of genetic diseases, history of hyperpyrexia, malignancy, autoimmune disorders, sexually transmitted diseases or inflammation of reproductive organs. Individuals who worked in high temperature environments or had occupational exposure to toxic chemicals or radiation were also excluded.

Semen samples were obtained by masturbation after 3–5 days of sexual abstinence and were allowed to liquefy within 30 min at 37°C. To harvest cell-free seminal plasma, we centrifuged seminal samples twice at 4°C. After the first centrifugation at 1,600 g for 10 min at 4°C, the supernatant was then centrifuged at 16,000 g for 10 min to remove cells and cell debris. The supernatant was carefully collected for subsequent assays.

### Stability Analysis of cfs-miRNA

In the following experiments of stability, semen plasma filtration, and Triton X-100 treatment, we focused on cfs-miRNA, because these have previously been performed on cfs-mRNA [19].

We left 5 seminal samples at room temperature for 0, 2, 4, 8, and 24 h. At each time point, 0.2 ml seminal plasma was subjected to RNA extraction for miRNA quantification. As a control for the effect of seminal RNases, we spiked purified cfs-miRNA from normozoospermic participants into seminal plasma of vasectomized individuals (n = 5). The amounts of testis-specific piRNAs, which should exist in seminal plasma of normozoospermia but not in the vasectomized individuals, were assessed.

### Filtration and Triton X-100 Treatment of Seminal Plasma

Seminal plasma from 5 healthy individuals was respectively divided into 5 aliquots: Two portions were individually passed through filters (Millipore) with pore sizes of 0.45, and 0.22 µm. One aliquot was added Triton X-100 to a final concentration of 1% (v/v) and incubated at room temperature for 20 min. The remaining two control aliquots were not subjected to filtration, but one of them was added Triton X-100 to a final concentration of 1% (v/v) after protection of RNA by the lysis reagent during RNA extraction. These aliquots of seminal plasma were subjected to RNA extraction for quantitative analysis of miRNAs. These methods have previously been described [19], [31].

### Preparation and Treatment for SMVs

As microvesicles are commonly smaller than 1.0 µm, cell-free seminal plasma was firstly passed through a 1.0 µm Protran Filter (Whatman) to remove any remaining macromolecules and debris. 0.4 ml of this filtrate was adjusted with 8.6 ml of phosphate-buffered saline (PBS) buffer (10 mM Na_2_HPO_4_, 1.8 mM KH_2_PO_4_, 50 mM NaCl, 2.7 mM KCl, pH 7.4) to a 9 ml final volume in a ultracentrifuge tube and spun at 100,000 g for 2 h in a SW 70 Ti Rotor at 4°C to pellet the SMVs. The final pellet, containing the SMVs, was then resuspended in sterile PBS and prepared for follow-up studies. The presence and characterization of SMVs was checked by transmission electron microscope.

Then, to disrupt any free RNA and RNA-binding proteins in the suspensions of SMVs, RNase A (Fermentas) was added to these suspensions at a final concentration of 100 µg/ml and incubated for 15 min at 37°C. After that, these suspensions were immediately treated with 0.25% (w/v) trypsin (Sigma) for 10 min at 37°C. Finally, the suspensions of SMVs were used for subsequent RNA isolation for mRNA and miRNA quantification.

### SMVs Filtration

As microvesicles was likely to be heterogeneous with a diameter of 30 nm–1.0 µm, suspensions of SMVs obtained from another group of 6 healthy participants were divided into 5 aliquots, respectively. Four aliquots were individually passed through 4 filters of different pore sizes (0.80, 0.45, 0.20 and 0.10 µm). The remaining aliquot was unfiltered. We then extracted the RNA from 0.4 ml of the filtered and unfiltered aliquots of SMVs suspensions for mRNA quantification.

### Filtration and Protease K treatment for Supernatant

The supernatant after ultracentrifugation may contain tiny microvesicles. Supernatants obtained from 4 healthy participants were divided into 2 aliquots, respectively. One was passed through a 0.10-µm filter. The remaining aliquot was unfiltered. Then RNA was extracted from 0.2 ml of the filtered and unfiltered aliquots for miRNA quantification.

As most of protein complexes should remain in the supernatant after ultracentrifugation, we added protease K (Fermentas) to the supernatant at a final concentration of 0.5% (w/v) and incubated for 0, 15, 30 min and 1, 2 h. At each time point, 0.2 ml supernatant was subjected to RNA extraction for miRNA quantification.

### RNA extraction

The seminal plasma, the suspension of SMVs, and the supernatant after ultracentrifugation were subjected to RNA extraction directly. Seminal plasma and SMVs were diluted to the same volume as the supernatant, for the experiments of comparisons between RNAs in seminal plasma, SMVs, and the supernatant. Different protocol of RNA extraction was applied for mRNA and miRNA assessment. The RNA extraction for mRNA quantification was performed as described previously [19] with minor modifications. Briefly, liquid for RNA isolation (0.4 ml) was mixed with 0.8 ml TRIzol LS (Invitrogen). The mixture was incubated at room temperature for 10 min. Then 0.22 ml of chloroform was added and mixed by vigorous shaking. After incubation at room temperature for 10 min, the mixture was centrifuged at 12,000 g for 15 min at 4°C. The aqueous layer was transferred to a new tube, mixed with isopropanol in a 1∶1 ratio and incubated at room temperature for 10 min. Then, the mixture was centrifuged at 12,000 g for 10 min at 4°C. The RNA pellet was dissolved in RNase-free water (0.1 ml) and then purified with an RNeasy Kit (Qiagen) according to the manufacturer's instructions. Total RNA was finally eluted with 20 µl of RNase-free water with RNase inhibitor (Fermentas).

As for the RNA extraction for miRNA quantification, 0.2 ml of fluid was mixed with 0.25 ml TRI-reagent buffer (0.15 NaCl, 1% SDS, 2.5 mM EDTA, 0.1 M Tris-HCl, pH 8.0). Then 9 mg PEG 20,000 (Sigma) was added to get a final concentration at 2% (w/v) to remove polysaccharides, which is very rich in seminal plasma. But for the suspension of SMVs, this step was skipped. The mixture was incubated for 10 min at room temperature, followed by centrifugation at 13,000 g for 10 min at 4°C. The aqueous layer was transferred to a new tube for the Trizol LS procedure according to the manufacturer's instruction. Briefly, the aqueous layer (0.4 ml) was mixed with 0.8 ml Trizol LS reagent and incubated for 5 min at room temperature and then centrifuged at 12,000 g for 10 min at 4°C to remove insoluble material. The cleared solution layer was transferred to a new tube and mixed with 0.22 ml chloroform. The mixture was incubated for 10 min at room temperature and centrifuged at 12,000 g for 15 min at 4°C and then the aqueous layer was transferred to a new tube. Equal volume of isopropyl alcohol was added to the aqueous layer, and the mixture was incubated for 10 min at room temperature and centrifuged at 12,000 g for 10 min at 4°C. The RNA pellet was washed with 75% ethanol. Finally, RNA was dissolved in 15 µl RNase-free water with RNase inhibitor.

To remove any contaminating DNA, DNase treatment [Recombinant DNase I (RNase-free); Takara] was carried out according to the manufacture's instructions.

### mRNAs and miRNAs Investigated

The genes that we selected were housekeeping gene (*ACTB*) and male reproductive organ-specific transcripts (*DDX4*, *PRM2*, *DEFB129*, *SERPINA5*, *TGM4*): (1) Housekeeping gene: β-actin (*ACTB*, NM_001101); (2) Testis-specific genes: including whole process spermatogenesis marker gene: DEAD (Asp-Glu-Ala-Asp) box polypeptide 4 (*DDX4*, NM_001317), and post-meiotic spermatogenic gene: Protamine 2 (*PRM2*, NM_002762); (3) Epididymis-specific genes: β-defensin 129 (*DEFB129*, NM_080831); (4) Seminal vesicle- specific genes: serpin peptidase inhibitor, clade A (alpha-1 antiproteinase, antitrypsin), member 5(*SERPINA5*, NM_000624); (5) Prostate-specific genes: transglutaminase 4 (*TGM4*, NM_003241). All primers were designed to span the intron. Information of these genes and primer sequence is given in supplemental data Table S1.

Two miRNAs (miR-34a, miR-141) existing widely in epithelial cells of male reproductive organs, two testis-specific miRNA (miR-202, miR-449a), and two piRNAs (piR-013423, piR-023386) were involved in this study. See supplemental data Table S2 for the information of probes and primers.

### cDNA Synthesis and qPCR for mRNAs

Reverse-transcription was performed for synthesizing cDNA with the RevertAidTM First Strand cDNA Synthesis Kit (Fermentas) according to the manufacturer's instruction.

Quantitative real-time PCR (qPCR) for mRNAs was performed as described previously [19] with an Mx3000P thermocycler (Stratagene) and with SYBR Green I used for detecting the fluorescence of amplified products. PCR conditions were: 95°C for 10 min (initial denaturation) and 40 cycles of 25 sec at 95°C (denaturation), 30 sec at the annealing temperature (Table S1), 30 sec at 72°C (elongation), and 8 sec at 81°C (fluorescence measurement). A melting curve was generated at the end of every run to ensure product uniformity. The gel-extracted PCR products were inserted in the pCR2.1-TOPO vectors (Invitrogen) and were subjected to DNA sequencing to confirm the correct amplification. To quantify these 6 different mRNAs, calibration curves were generated by use of serial dilutions of the plasmid DNA.

### cDNA Synthesis and qPCR for miRNAs

The quantity of miRNAs was determined using qPCR based on enzymatic stem-loop probes ligation as described previously [32], which has been shown to be highly specific for the mature miRNA. Briefly, for cDNA synthesis, total RNA was mixed with probes (20 fmol) and incubated at 65°C for 3 min on a Gradient thermal cycler (Eppendorf), then slowly cooled to room temperature (about 30 min), and finally placed on ice for the formation of nicked heteroduplex structures. Enzymatic ligation was performed on the thermal cycler by incubation at 22°C for 1 h and 65°C for 10 min with the presence of T4 DNA ligase (Fermentas), DNA ligation buffer, PEG 4000, and RNase inhibitor in a final volume of 20 µl.

Real-time PCR was performed with the Mx3000P thermocycler. The ligation product (2 µl) was pipetted as PCR templates into DimerEraser SYBR Green Mastermix (Takara) to obtain a 20 µl PCR reaction. The reactions were incubated in a 96-well plate at 95°C for 30 sec, followed by 40 cycles of 95°C for 5 sec, 60°C for 20 sec, and 72°C for 20 sec. To ensure product uniformity, a melting curve was generated at the end of every run, and the PCR product was analyzed on an 8% polyacrylamide gel (length: 26 cm). Calibration curves were constructed by use of serial dilutions of synthetic miRNA with known concentration.

### Statistic Analysis

Statistical analyses were performed using SigmaStat 2.03 software (SPSS Inc.). The Friedman test was used for analyzing the stability of cfs-miRNA, proportions of RNAs that included within SMVs, and for experiments of Triton X-100 treatment and all filtrations. When this test gave significant results, it was followed by Student–Newman–Keuls (SNK) test. *P*<0.05 was considered as significant.

## Results

### Stability of cfs-miRNA

The stability of cfs-miRNA was evaluated by a time-course (0, 2, 4, 8, and 24 h, at room temperature) analysis of 4 miRNAs and 2 piRNAs involved in this study. All these cfs-miRNAs were stable at least within 24 h. We observed no significant degradation of any miRNA at any of these serial time points (supplemental data Figure S1; *P*>0.05, SNK test). While in the spiked control, exogenous RNA (contain piRNA from testis) obtained from seminal plasma of healthy individuals was added into the seminal plasma of vasectomized participants (do not contain piRNA); Only very small amount (11.7% for piRNA-013423, 12.3% for piRNA-023386) of piRNA remained after 2 min incubation at room temperature, confirming the RNase activity in semen.

### Most cfs-miRNA Remained after Filtration or Triton X-100 Treatment

**Figure 1:**
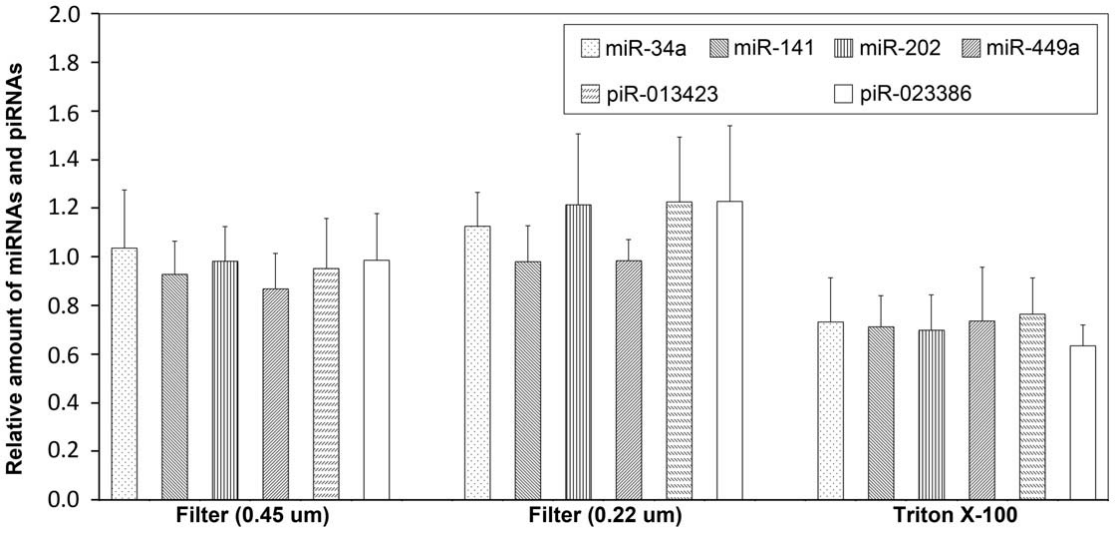
The effect of filtration and Triton X-100 on the amount of cfs-miRNAs. Seminal plasma was filtered through 0.45-, and 0.22-µm pores, or incubated with 1% (v/v) Triton X-100 at room temperature for 20 min. The amount of miRNAs was normalized to the unfiltered aliquot. Each column represents the mean of 5 independent samples. Error bars indicate the SD.

Filtration and Triton X-100 treatment were taken as the simple and preliminary methods to provide information of the complexes the cfs-miRNA were bound with. The clearly reductive effects of these treatments on cfs-mRNA have been observed in our previous study [19]. But for cfs-miRNA, after filtrating seminal plasma through 0.45-, or 0.22-µm pores, no significant change of the amount of any miRNA was observed (Figure 1; *P*>0.05, SNK test). After incubation with 1% (v/v) Triton X-100 at room temperature for 20 min, although significant degradation of miRNA in seminal plasma was observed (Figure 1; *P*<0.05, SNK test), the main part of them remained detectable. The mean percentages of remained miRNA varied from 63.4% (piR-023386) to 75.8% (piR-013423). In contrast, only 2.3% of *ACTB* and no *DDX4* mRNAs were detected under the same incubation [19].

### Isolation and Characterization of SMVs

**Figure 2:**
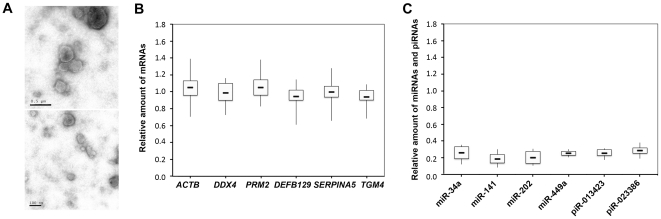
Characterization of SMVs and the proportions of cfs-mRNAs and cfs-miRNAs in SMVs. SMVs (A) were approximately 30 nm-0.5 µm in diameter using a transmission electron microscope. The amount of mRNAs (B, n = 16) and miRNAs (C, n = 6) in the SMVs was normalized to the amount in the seminal plasma. Horizontal lines within the boxes denote the median. The boxes and whiskers represent intervals between the 25th to 75th percentiles, and the 2.5th to 97.5th percentiles, respectively.

The existence of significant amounts of SMVs in human seminal plasma, and the very different reductive effect of filtration and Triton X-100 on cfs-mRNA [19] and cfs-miRNA (Figure 1) prompted us to isolate the SMVs. The SMVs were isolated successfully in all samples. Under transmission electron microscope, these SMVs are primarily 30 nm-0.5 µm in diameter (Figure 2A).

### cfs-mRNA in SMVs with Different Size

We then further evaluated the proportions of cfs-mRNA and cfs-miRNA that presents in the form of SMVs. The RNA in the seminal plasma and its SMVs suspension was extracted for comparing the amounts of 6 mRNAs, 4 miRNAs, and 2 piRNAs by qPCR.

In initial experiments (unpublished data), we observed that the amounts of cfs-mRNA in seminal plasma were almost the same as in the SMVs. To confirm this interesting result, seminal samples from 16 healthy participants were analyzed. Overall, no obvious change in *ACTB*, *DDX4*, *PRM2*, *DEFB129*, *SERPINA5* and *TGM4* mRNA levels between concentrations of total cfs-mRNA in seminal plasma and the part of SMVs-sequestered mRNA was observed (Figure 2B, Table S3; n = 16, *P* >0.05, SNK test). As for the mRNA in the supernatant, qPCR failed or gave a high threshold cycle value in most cases. Thus, SMVs- sequestered mRNA constituted the majority of cfs-mRNA.

**Figure 3:**
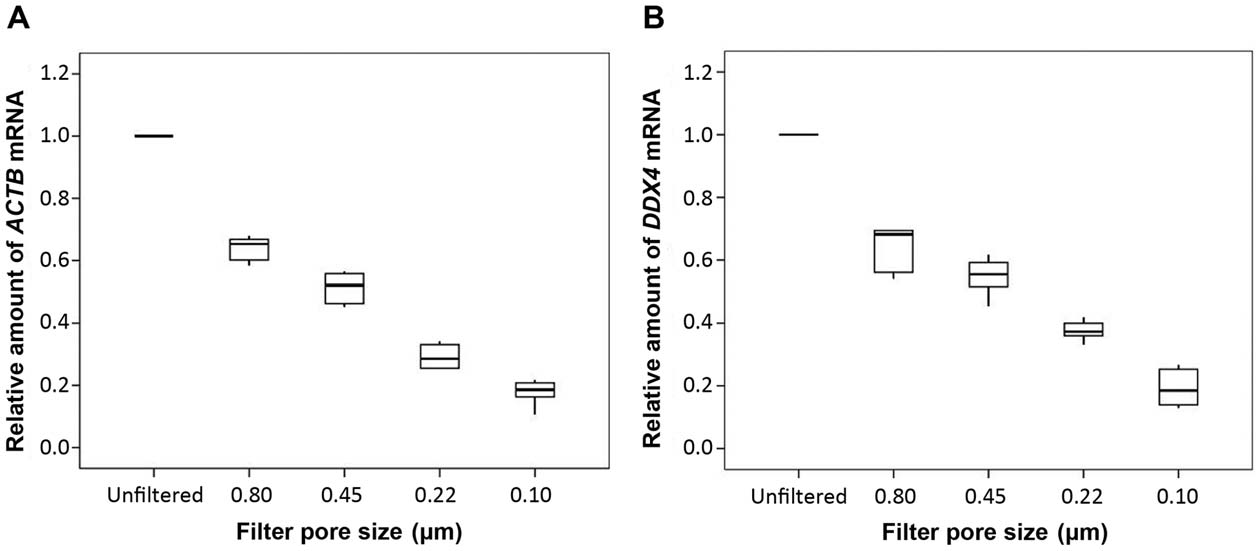
The representative result of heterogeneous in quantity of cfsRNA according to size of SMVs. SMVs suspensions from 6 healthy participants were filtered through 0.80, 0.45, 0.22 and 0.10 µm pores. The amount of mRNAs at each pore size was normalized to the unfiltered aliquot, respectively. Horizontal lines within the boxes denote the median. The boxes represent the interval between the 25th and 75th percentiles, and the whiskers represent the interval between the 2.5th and 97.5th percentiles.

As microvesicles was likely to be heterogeneous in size [33]–[35], the SMVs- sequestered nature of cfs-mRNA was further investigated by filtrating SMVs through filters with different pore sizes (0.80, 0.45, 0.20, and 0.10 µm). To exclude the possibility that these filters bind RNA nonspecifically, we also filtered purified RNA through filters with different size pores and recovered more than 85% of these 6 mRNAs. Clearly observable concentration reductions in each transcript after 0.80, 0.45, 0.20 and 0.10-µm filtration step were observed (Figure 3, Table S4; *P*<0.01, SNK test).

In contrast, only small part of cfs-miRNA was detected in the SMVs (Figure 2C), with median percentage (relative to seminal plasma; n = 6) varying from 18.4% (miR-141) to 28.5% (piR-023386). The proportions of SMVs- sequestered miRNAs were not related with different organs.

### Many cfs-miRNAs were Associated with Protein

**Figure 4:**
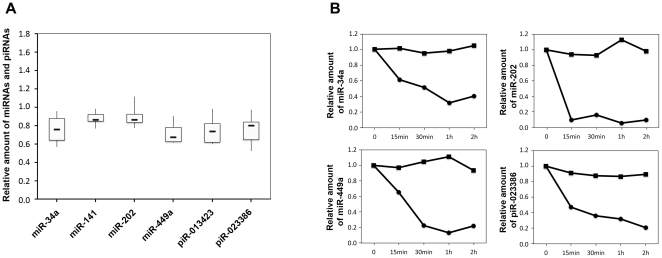
Most cfs-miRNAs remained in the supernatant and were dramatically degraded by intense protease K digestion. The amount of miRNAs in the supernatant (A) was normalized to the seminal plasma. Horizontal lines within the boxes denote the median (n = 6). The boxes and whiskers represent intervals between the 25th to 75th percentiles, and the 2.5th to 97.5th percentiles, respectively. For protease K digestion (B), at each time point, the supernatant incubated with 0.5% (w/v) protease K (circle dots) or not (square dots) was subjected to miRNA quantification. The amounts at time 0 were taken as 1.0.

Because only a small part of cfs-miRNA was found in the SMVs, so we determined the proportion of miRNA in the supernatant after ultracentrifugation for SMVs recovery. The major part of cfs-miRNA existed in the supernatant, with median percentage (relative to seminal plasma; n = 6) varying from 67.2% (piR-013423) to 86.5% (miR-141) (Figure 4A).

We continued to explore the existing form of the majority of cfs-miRNA in the supernatant. The supernatant was filtered through the filter with the 0.10- µm pore, through which it is hard to filter the seminal plasma directly because of the viscosity. Almost all of cfs-miRNA passed the filter (Supplementary data Figure S2). Even in some cases, a light increase was observed in the filtrate.

Considering that abundant proteins exist in the seminal plasma, and most of them should remain in the supernatant, we then digested the supernatant with protease K at a high concentration (0.5%, w/v). Generally, within 15 min, all cfs-miRNA we assessed were significantly degraded in the presence of protease K, while remained stable in the untreated control. The representative results of some cfs-miRNAs from one sample were shown in Figure 4B.

## Discussion

To reveal the physical nature of cell-free RNAs can provide insight into mechanisms accounting for their stability and intercellular communication. Here we found very different forms between the existence of cfs-mRNA and cfs-miRNA, and discussed the implications for their origin, function and developing them as non-invasive biomarkers.

Our results on cfs-mRNAs revealed that almost all of them existed in SMVs. This result was confirmed in 16 healthy individuals, without any significant differences between the amount of cfs-mRNAs we quantified in the seminal plasma and in the SMVs. All these 6 different genes that specifically expressed in each male reproductive organ showed the same result. Previous results [19] also showed the significant degradation of cfs-mRNA by incubating seminal plasma with Triton X-100, which could enhance the permeability of the SMVs membrane and expose RNA to RNase in seminal plasma. Therefore, these data demonstrated cfs-mRNA was mainly contained inside SMVs and was thereby protected from external RNases by the surrounding membrane. As for cell-free mRNAs in other body fluids, previous report [36], [37] on circulating mRNAs suggested that some of them were contained inside microvesicles.

As for the cfs-miRNA, we first demonstrated its stability at least within 24 h at room temperature, which apparently cannot be explained by the intrinsic stability of miRNA [38]. Our results indicated that small vesicles contribute minimally to the concentration of cfs-miRNAs. First, only around 20% of cfs-miRNA was contained inside SMVs, as demonstrated by the results of Triton X-100 treatment and the cfs-miRNA proportion in SMVs. Second, no significant change of the amount of any miRNA was observed after filtration seminal plasma through 0.45-, or 0.22-µm pores, suggesting the sizes of these SMVs-sequestered cfs-miRNAs were below 0.22 µm. But the possibility cannot be excluded that few of them existed in SMVs above 0.22-µm, while filtration may enhance the efficacy of RNA extraction.

Further experiments on the cfs-miRNAs in the supernatant reveal that most of cfs-miRNA was bound with proteins. Almost all cfs-miRNA in the supernatant passed through the 0.10-µm filter. Dramatical degradation of cfs-miRNA in the supernatant was observed after the 15-min incubation with 0.5% (w/v) protease K, and only trace amounts of them remained after the 30-min incubation. Some previous studies revealed the existence of miRNAs in membrane-bound microvesicles acquired from the cell-culture medium [16], [39] or body fluids [40], [41], and lead to the dominant hypothesis that vesicles, as carriers of cell-free miRNAs, protect them against degradation. However, recent reports [42], [43] demonstrated most circulating miRNAs are associated Ago2 protein, a functional protein of miRNA-induced silencing complex. Also, recent results [44] indicate many circulating miRNA in plasma existed in platelets, which may overwhelm the disease-, or tissue-specific signature if were not removed. Our results lead us to believe that most cfs-miRNA was released and protected from degradation in the form of miRNA-induced silencing complex, while only small proportion in the form of SMVs.

SMVs are regarded as very stable ultrastructural organizations and vehicles for intercellular communication, which should contribute to the stability, origin, and possible function of extracellular RNAs in semen.

Although coherent similar results were observed for these different tissue-specific cfs-mRNAs and cfs-miRNAs, the possible origin of SMVs from these organs should be different. Exosomes and shedding vesicles from epithelium of epididymis, prostate, and seminal vesicle [26]–[28], [35], [45], [46] may be responsible for the SMVs-sequestered cfs-mRNA and cfs-miRNA through apocrine secretion or other mechanism. As for the testis-specific mRNAs and miRNAs, although most of large and medium-sized sperm residual body of apoptotic germ cells were phagocytized [47], some smaller shedding vesicles might be redundant cargoes loading cellular RNA in luminal fluids of testis and epididymis. In testis, RNA binding proteins can stabilize cellular mRNAs transcribed before mid-spermatogenesis and store them for translation during spermiogenesis period, during which *de novo* transcription is halted permanently [48], [49]. But out results suggest that this kind of protection do not contribute to the large amounts of cfs-mRNAs. We also observed that the amounts of SMVs-sequestered cfs-mRNA were highly variable depending on the different sizes of SMVs (Figure 3). As microvesicles <0.10 µm were identified as exosomes [33], [34], our results showed the amounts of cfs-mRNA were richer inside shedding vesicles (usually their sizes are 0.10–1 µm [34]), which could be the microparticles and/or the apoptotic blebs [34].

On the other hand, many investigations [14]–[18] have proposed extracellular mRNA and miRNA as means of intercellular communications by vesicle-mediated transfer between cells. It is tempting to think that extracellular RNAs in luminal fluids of male reproductive organs could commute between cells and attribute to the gene expressions. Indeed, delivering molecules by microvesicles have been demonstrated in male reproductive system. For instance, one molecular mechanism for sperm maturation is delivering proteins into sperm by epididymosome [50]–[52]; SMVs or prostasome was also found to attribute to sperm function by delivering molecules into sperm [53]–[55]. Our previous results [19] demonstrated that cfs-mRNA contained intact transcripts, which could be template for protein translation.

This finding may contribute to the strategy of developing cfs-mRNA and cfs-miRNA as biomarkers of male reproductive system. Seminal plasma may be the most complicated type and particularly challenging for RNA extraction among all human body fluids, due to high level of proteins, polysaccharides, and other secondary metabolites. But harvesting SMVs for cfs-mRNA recovery by routine methods could be a simple and better alternative because it avoids the influence of proteins, polysaccharides, and other compounds. Also, there are many kinds of methods for protein fractionation, enrichment, concentrating, and recovery, through which the protein complexes could be collected for recovering most amounts of cfs-miRNA. Another issue is the existence of physical barrier between blood and male reproductive system, such as the blood–testis barrier and blood–epididymis barrier. Cell-free mRNAs and miRNAs from testis or epididymis may be hard to be detected in blood, given that they were enclosed in the microvesicles or bound with protein complexes, which should not pass these barriers.

In conclusion, this work demonstrated that most cfs-mRNA was contained inside SMVs, while the majority of cfs-miRNA was bound with protein complexes. Our data shed light on the strategy of developing cfs-mRNA and cfs-miRNA as biomarkers of male reproductive system. Microvesicles have been shown to transfer proteins and RNA from cell to cell and play a role in intercellular communication. Whether the SMVs or the protein-sequestered cfs-mRNA or cfs-miRNA, which is stored in male reproductive organs before ejaculation, has function in male reproduction need further investigation.

## Supporting Information

Figure S1 Stability of cfs-miRNAs and piRNAs. Seminal plasma samples of normozoospermic individuals were incubated at room temperature up to 24 h. Amounts of miRNAs and piRNAs were measured by real time PCR at each time point and normalized to the amount at time 0. The last two spiked piRNAs were measured after adding the recovered cfs-miRNA into seminal plasma of vasectomized participants, which should not contain piRNAs. Each column represents the mean of 5 independent samples. Error bars indicate the SD.
(TIF)

Figure S2 The amount of miRNAs in the supernatant was not affected by filtration through the 0.10-µm filter. Each dot represents the amount of miRNA after filtration (relative to the unfiltered aliquot).
(TIF)

Table S1 Specific primers used for RT-PCR of mRNAs.
(DOC)

Table S2 Ligation probes and PCR primers for miRNAs.
(DOC)

Table S3 Median levels of gene mRNAs in cell-free seminal RNA and SMVs.
(DOC)

Table S4 Relative mRNA concentrations in SMVs filtered through different sizes pores.
(DOC)
