# Supplementary figures and images for: Cell-Free Seminal mRNA and MicroRNA Exist in Different Forms
Source: PLoS One. 2012 Apr 10;7(4):e34566. doi: 10.1371/journal.pone.0034566 (PMC3323549; doi:10.1371/journal.pone.0034566)

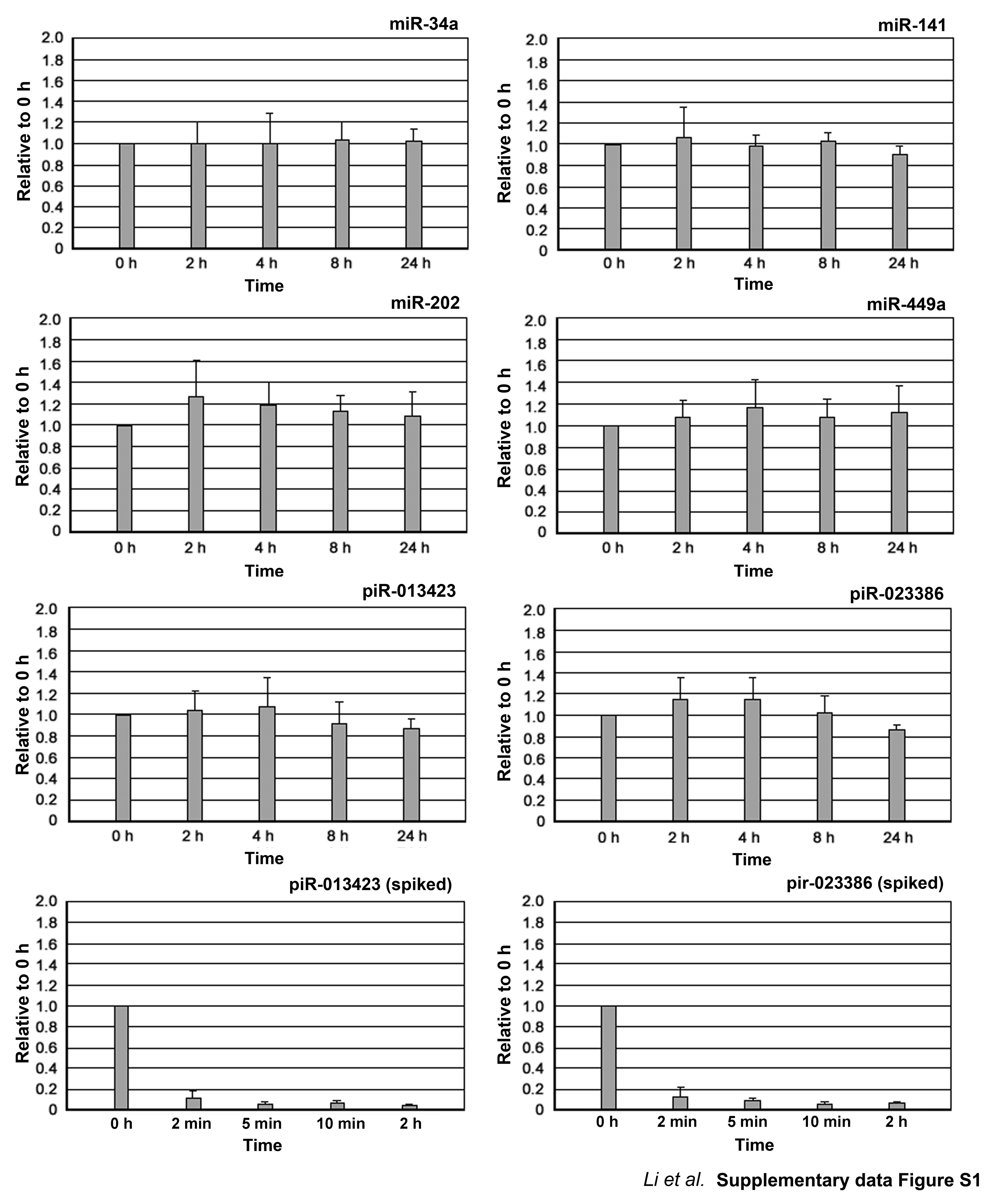

Supplement: Figure S1 — Stability of cfs-miRNAs and piRNAs. Seminal plasma samples of normozoospermic individuals were incubated at room temperature up to 24 h. Amounts of miRNAs and piRNAs were measured by real time PCR at each time point and normalized to the amount at time 0. The last two spiked piRNAs were measured after adding the recovered cfs-miRNA into seminal plasma of vasectomized participants, which should not contain piRNAs. Each column represents the mean of 5 independent samples. Error bars indicate the SD. (TIF) [file pone.0034566.s001.tif]

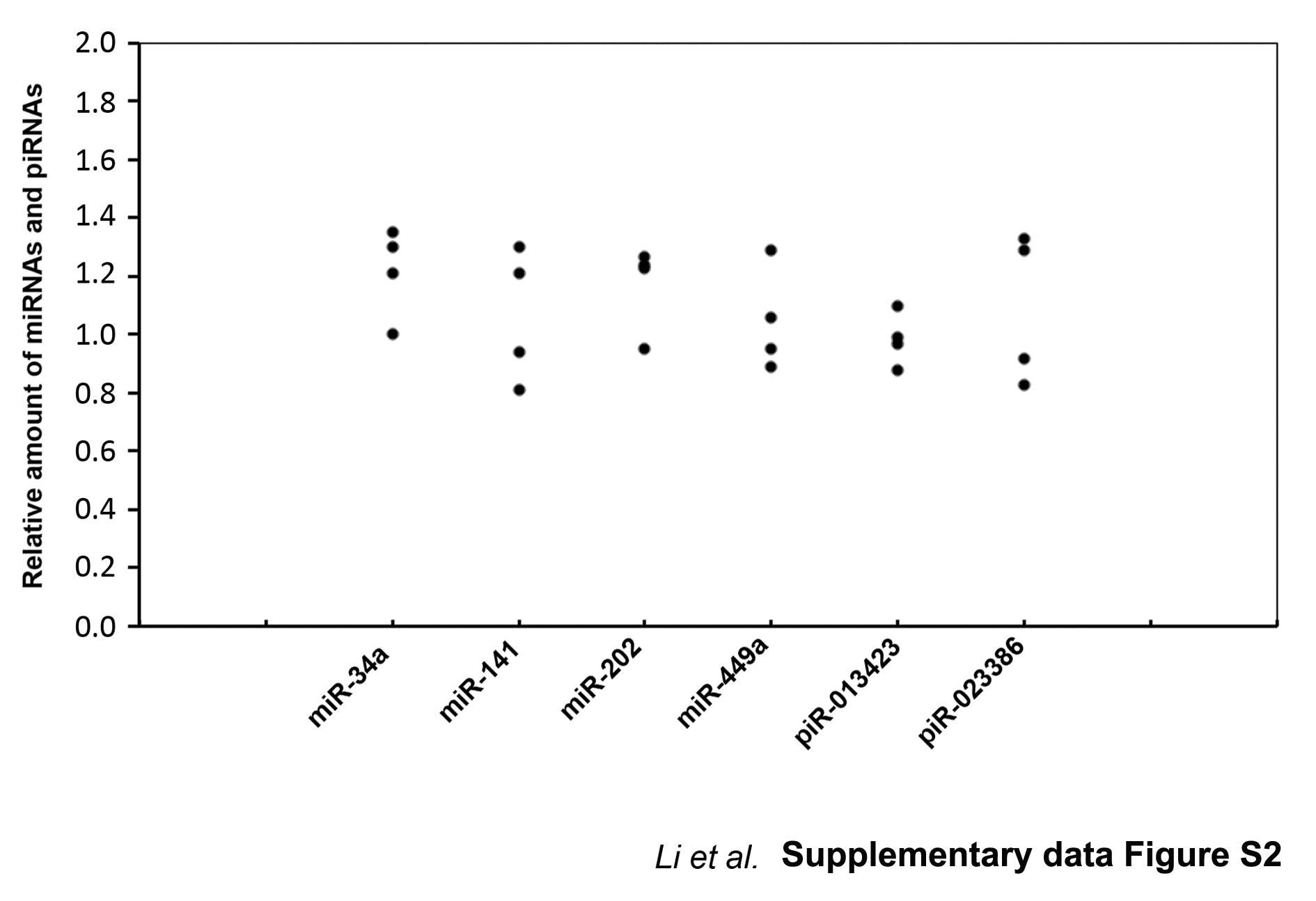

Supplement: Figure S2 — The amount of miRNAs in the supernatant was not affected by filtration through the 0.10-µm filter. Each dot represents the amount of miRNA after filtration (relative to the unfiltered aliquot). (TIF) [file pone.0034566.s002.tif]
