# Supplementary material for: Cell-Free Seminal mRNA and MicroRNA Exist in Different Forms
Source: PLoS One. 2012 Apr 10;7(4):e34566. doi: 10.1371/journal.pone.0034566 (PMC3323549; doi:10.1371/journal.pone.0034566)
Supplement: Table S1 — Specific primers used for RT-PCR of mRNAs. (DOC) [file pone.0034566.s003.doc]

| **Table S1.** Specific primers used for RT-PCR of mRNAs. | | | | | |
| --- | --- | --- | --- | --- | --- |
| **Gene symbol** | **Gene name (GeneBank)** | **Origin tissue** | **Primer sequences*^a^*** | **AT*^b^*(°C)** | **Amplicon**  (bp) |
| *ACTB* | β-actin (NM_ 001101) | housekeeping | F: GGCATGGGTCAGAAGGATT  R: AGGTGTGGTGCCAGATTTTC | 63 | 133 |
| *DDX4* | DEAD box polypeptide 4 (NM_ 001317) | testis (germ cell) | F:TTCTTGTGGAAGTGTCTGGACA  R: CGACTGGCAGTTATTCCATCA | 63 | 236 |
| *PRM2* | protamine 2 (NM_ 002762) | testis (post-meiotic) | F:AGAGGACCCATGGCCAGTCT  R:TCCTGGTTCTGCAGCCTCTG | 57 | 153 |
| *DEFB129* | β-defensin 129 (NM_ 080831) | epididymis | F: TATCTTTGCCAGCCTCAT  R: CCATATTGCAGGTAGTTT | 57 | 192 |
| *SERPINA5* | serpin peptidase inhibitor, clade, member 5 (NM_ 000624) | seminal vesicle | F: GATAGCAATGCGGTCGTGAT  R: GGTTCCGGTCCAGGAGGTAG | 57 | 175 |
| *TGM4* | transglutaminase 4 (NM_003241) | prostate | F: GCATTTCCCTGCTGACTG  R: AGCCTGCTTCGTGTTGTAG | 57 | 212 |
| ***^a^*** 5' to 3' sequence, F: Forward primer; R: Reverse primer.  ***^b^*** Annealing temperature. | | | | | |
