# Supplementary material for: Cell-Free Seminal mRNA and MicroRNA Exist in Different Forms
Source: PLoS One. 2012 Apr 10;7(4):e34566. doi: 10.1371/journal.pone.0034566 (PMC3323549; doi:10.1371/journal.pone.0034566)
Supplement: Table S2 — Ligation probes and PCR primers for miRNAs. (DOC) [file pone.0034566.s004.doc]

| **Table S2.** Ligation probes and PCR primers for miRNAs. | | |
| --- | --- | --- |
| **miRNAs** | **Probe and primer sequences*^a^*** | **Amplicon**  (bp) |
| hsa-miR*-*34a | Prob 1: GGTATCCAGGGAAGTGGATACGAAGAGCTCACACAACCAGCTA  Prob 2: PO4-AGACACTGCCAGCCTGTCAATTAGTCGTACAGGTATTACGACA  F: GGGAAGTGGATACGAAGAG  R: TACGACTAATTGACAGGCTG | 64 |
| hsa-miR*-*141 | Prob 1: GGTCTCCAGGGAAGTCCTCACGAACGTAACAGCCATCTTTACC  Prob 2: PO4-AGACAGTGTTA GCGTGTCAGTTAGTGGTACAGGTATTACGACA  F: CAGGGAAGTCCTCACGAA  R: CACTAACTGACACGCTAACA | 63 |
| hsa-miR*-*202 | Prob 1: GGTCTCCAGGGAAGTCGTCACGAACGCATCTAA TTCCCATGCC  Prob 2: PO4-CTATACCTCTGCCTATAACTTAGTGCCACAGGTATTACGACA  F: GTCTCCAGGGAAGTCGTC  R: CACTAAGTTATAGGCAGAGGT | 68 |
| hsa-miR*-*449a | Prob 1: GGTCAGGTCGGAAGTCCTCACGAACGTTACTGTACCAGCTAACA  Prob 2: PO4-ATACACTGCCAGCGTGTAACTTAGTGCCACAGGTAATACGACA  F: AGGTCGGAAGTCCTCACG  R: CGGTCGCACATTGAATCAC | 66 |
| hsa-piR*-* 013423 | Prob 1: GGTATCCAGGGAAGTGGATACGAAGTGTAAGGCAATCAGTCTTACT  Prob 2: PO4-TGTTGCAGACAGCCACGGACAACGCAACCAATTAGTCGTACAGGTATTACGACA  F: GGGAAGTGGATACGAAGTG  R: TACGACTAATTGGTTGCGTTG | 78 |
| hsa-piR*-* 023386 | Prob 1: GGTCTCCAGGGAAGTGCTCAGCTACTCGTGCCGACGCCTCCACGAG  Prob 2: PO4-TGTCTTCTCGTTCCCACAGACAGTTAGTGGTACAGGTATTACGACA  F: GTCTCCAGGGAAGTGCTC  R: CTAACTGTCTGTGGGAACGA | 72 |
| ***^a^*** 5' to 3' sequence, F: Forward primer; R: Reverse primer. | | |
