# Supplementary material for: Cell-Free Seminal mRNA and MicroRNA Exist in Different Forms
Source: PLoS One. 2012 Apr 10;7(4):e34566. doi: 10.1371/journal.pone.0034566 (PMC3323549; doi:10.1371/journal.pone.0034566)
Supplement: Table S3 — Median levels of gene mRNAs in cell-free seminal RNA and SMVs. (DOC) [file pone.0034566.s005.doc]

| **Table S3.** Median levels of gene mRNAs in cell-free seminal RNA and SMVs*^a^* (ng/mL) | | | | |  | |
| --- | --- | --- | --- | --- | --- | --- |
| **Gene** | **cell-free seminal RNA** | **SMVs** |  |  | |  |
|  |  | |  |
| *ACTB* | 839.5  (755.6–963.3) | 841.7  (786.9–1089.8) |  |  | |  |
| *DDX4* | 6.05  (5.42–7.08) | 6.24  (5.17–6.45) |  |  | |  |
| *PRM2* | 335.1  (301.8–394.4) | 363.8  (293.0–417.2) |  |  | |  |
| *DEFB129* | 1.18  (0.84–1.28) | 1.03  (0.69–1.28) |  |  | |  |
| *SERPINA5* | 1.30  (1.25–1.62) | 1.32  (1.19–1.61) |  |  | |  |
| *TGM4* | 373.0  (311.1–504.5) | 333.3  (286.2–422.7) |  |  | |  |
| ***^a^*** The interquartile ranges were shown in brackets (n=16). | | | | |  | |
