# Supplementary material for: Cell-Free Seminal mRNA and MicroRNA Exist in Different Forms
Source: PLoS One. 2012 Apr 10;7(4):e34566. doi: 10.1371/journal.pone.0034566 (PMC3323549; doi:10.1371/journal.pone.0034566)
Supplement: Table S4 — Relative mRNA concentrations in SMVs filtered through different sizes pores. (DOC) [file pone.0034566.s006.doc]

| **Table S4.** Relatived mRNA concentrations in SMVs filtered through different sizes pores. | | | | | | |
| --- | --- | --- | --- | --- | --- | --- |
| **Gene** |  | **Relatived median levels of mRNAs in SMVs filtered through different sizes pores (*μ*m)*^a^*** | | | | |
|  |  | **unfiltered** | **0.80** | **0.45** | **0.20** | **0.10** |
| *ACTB* |  | 1 | 0.65 | 0.52 | 0.29 | 0.20 |
| *DDX4* |  | 1 | 0.64 | 0.55 | 0.38 | 0.19 |
| *PRM2* |  | 1 | 0.76 | 0.50 | 0.41 | 0.34 |
| *DEFB129* |  | 1 | 0.89 | 0.57 | 0.33 | 0.22 |
| *SERPINA5* |  | 1 | 0.62 | 0.41 | 0.37 | 0.31 |
| *TGM4* |  | 1 | 0.82 | 0.50 | 0.32 | 0.20 |
| ***^a^*** The amounts of cfsRNA were normalized to their unfiltered aliquot. | | | | | | |
